# A Decade of Veteran Voices: Examining Patient Portal Enhancements Through the Lens of User-Centered Design

**DOI:** 10.2196/10413

**Published:** 2018-07-10

**Authors:** Kim M Nazi, Carolyn L Turvey, Dawn M Klein, Timothy P Hogan

**Affiliations:** ^1^ Veterans and Consumers Health Informatics Office, Office of Connected Care Veterans Health Administration US Department of Veterans Affairs Coxsackie, NY United States; ^2^ Iowa City VA Health Care System Comprehensive Access and Delivery Research and Evaluation Center Iowa City, IA United States; ^3^ Department of Psychiatry The University of Iowa Carver College of Medicine Iowa City, IA United States; ^4^ Center for Healthcare Organization and Implementation Research (CHOIR) Edith Nourse Rogers Memorial Veterans Hospital Bedford, MA United States; ^5^ Division of Health Informatics and Implementation Science Department of Quantitative Health Sciences University of Massachusetts Medical School Worcester, MA United States

**Keywords:** patient portal, user-centered design, eHealth, veteran

## Abstract

**Background:**

Health care systems have entered a new era focused on patient engagement. Patient portals linked to electronic health records are recognized as a promising multifaceted tool to help achieve patient engagement goals. Achieving significant growth in adoption and use requires agile evaluation methods to complement periodic formal research efforts.

**Objective:**

This paper describes one of the implementation strategies that the Department of Veterans Affairs (VA) has used to foster the adoption and sustained use of its patient portal, My Health*e*Vet, over the last decade: an ongoing focus on user-centered design (UCD). This strategy entails understanding the users and their tasks and goals and optimizing portal design and functionality accordingly. Using a case study approach, we present a comparison of early user demographics and preferences with more recent data and several examples to illustrate how a UCD can serve as an effective implementation strategy for a patient portal within a large integrated health care system.

**Methods:**

VA has employed a customer experience analytics (CXA) survey on its patient portal since 2007 to enable ongoing direct user feedback. In a continuous cycle, a random sample of site visitors is invited to participate in the Web-based survey. CXA model questions are used to track and trend satisfaction, while custom questions collect data about users’ characteristics, needs, and preferences. In this case study, we performed analyses of descriptive statistics comparing user characteristics and preferences from FY2008 (wherein “FY” means “fiscal year”) to FY2017 and user trends regarding satisfaction with and utilization of specific portal functions over the last decade, as well as qualitative content analysis of user’s open-ended survey comments.

**Results:**

User feedback has guided the development of enhancements to core components of the My Health*e*Vet portal including available features, content, interface design, prospective functional design, and related policies. Ten-year data regarding user characteristics and portal utilization demonstrate trends toward greater patient engagement and satisfaction. Administration of a continuous voluntary Web-based survey is an efficient and effective way to capture veterans’ voices about who they are, how they use the patient portal, needed system improvements, and desired additional services.

**Conclusions:**

Leveraging “voice-of-the-customer” techniques as part of patient portal implementation can ensure that such systems meet users’ needs in ways that are agile and most effective. Through this strategy, VA has fostered significant adoption and use of My Health*e*Vet to engage patients in managing their health.

## Introduction

### Background

Health care systems have entered a new era focused on patient engagement [1-3] described by enthusiasts as “the holy grail of health care” [4] and the “blockbuster drug of the century” [5]. Patient engagement strategies are designed to empower patients to play a more active role in their health care and make informed decisions, improve the patient experience, increase patient satisfaction, and achieve better health outcomes. Patient portals linked to electronic health records (EHRs) are recognized as a promising multifaceted tool to help achieve these patient engagement goals [6-9]. However, the adoption and sustained use of portals has generally fallen short of initial optimism [10-13] even in light of the significant growth in EHRs and tethered patient portals incentivized by Meaningful Use [14]. Positive benefits of portal use have been demonstrated [15-19], and the OpenNotes movement [20] has promoted patient engagement through health records transparency by enabling patient access to provider notes. Evidence indicates that such access improves communication and trust, patient safety, and, potentially, patient outcomes [21-25]. Two large integrated health care systems that launched tethered patient portals in 2003 with significant patient adoption and sustained use are Kaiser Permanente (KP) and the Department of Veterans Affairs (VA). KP’s portal, My Health Manager, is used by more than 5 million members, representing about 70% of adult KP members [26]. VA’s patient portal, My Health*e*Vet, has more than 4 million registered users (69% of VA patients receiving health care services in FY 2017 [wherein “FY” means “fiscal year”]), with 2.5 million authenticated Premium accounts (42% of VA patients receiving health care services) required for access to all portal features [27]. To better understand what elements are driving this adoption and sustained use, an implementation case study approach is warranted. One of the implementation strategies that are critical to foster the adoption and sustained use of patient portals is an ongoing focus on user-centered design (UCD). This is often accomplished as part of periodic research studies; however, more timely and agile methods are needed to design and evaluate patient portals.

### User-Centered Design

UCD is a design philosophy and evaluation process that focuses on the end user’s characteristics, needs, preferences, and limitations throughout the design process and development lifecycle [28]. The emphasis of UCD is on understanding the end users and their tasks and goals and optimizing the product to enable the users to fulfill these, rather than requiring users to adapt to the designer’s preferences [29]. UCD of eHealth applications, such as patient portals, necessitates ongoing assessment of user characteristics and preferences and incorporation of assessment insights into ongoing portal development and enhancements. This process includes focusing on what features are considered to be most essential by users [30]. Published compilations of implementation strategies have called for the further development of processes like UCD as a means to obtain and use patient or consumer feedback to support the adoption of innovations and practice change efforts in health care and other settings [31].

VA has used various methods over the last decade to achieve UCD for My Health*e*Vet; among them, the principal method has been a continuous, voluntary, and anonymous survey of end users. As a complement to periodic formal research studies [32,33], this ongoing assessment offers the advantage of rapid continuous feedback, which is part of a cyclical process for improvement that entails understanding users, eliciting their input, identifying changes or future design implications, deploying enhancements, and then obtaining feedback to evaluate these enhancements. This method enables VA to obtain ongoing direct feedback from veterans, which can then be leveraged to improve the patient experience.

### About the Department of Veterans Affairs’ Patient Portal

VA is the largest integrated health care system in the United States and has been a pioneer in enabling patients to access and download their VA medical record data using the Blue Button feature [34,35]. This includes OpenNotes, which are known in My Health*e*Vet as VA Notes and contain both clinical and mental health providers’ notes [36]. The My Health*e*Vet patient portal [37] is tethered to the VA EHR and provides a suite of Web-based tools. Veterans self-register to create a basic account and can then self-enter information into their personal health record and access health education resources. VA patients who are matched by the system via the Master Veteran Index are automatically upgraded to an Advanced account and can request VA prescription refills. Patients who complete a one-time process of identity authentication (in person or Web-based) are upgraded to a Premium account and can then access all portal features, including access to health record information and Secure Messaging with VA health care professionals.

Use of My Health*e*Vet continues to grow. In fiscal year (FY)2017, portal user activity demonstrated significant increases compared with that in FY2016, including a 20.7% increase in Web-based prescription refills, a 33.9% increase in Secure Messaging exchanges between VA patients and their health care team, and a 38.7% increase in use of the VA Blue Button feature [27].

In this paper, we examine one of the implementation strategies that VA has used to foster adoption and sustained use of its patient portal over the last decade: an ongoing focus on UCD. This includes iterative use of survey and operational data with user interface redesign to meet the needs and preferences of veteran users. We describe the organization’s implementation strategy for agile UCD and present unique 10-year data on user adoption, characteristics, and utilization to demonstrate trends toward greater patient engagement and satisfaction. Following an initial analysis of portal users and their preferences in 2007 [38], we compared the characteristics of patient portal users one decade later and used a case study approach to present several examples of how user preferences and continuous feedback have informed the evolution of VA’s patient portal.

## Methods

Since 2007, VA has used the ForeSee customer experience analytics (CXA) survey tool for the direct measurement of customer satisfaction and prioritization of enhancements. The CXA survey is a standardized method of measuring and monitoring customer satisfaction based on the American Customer Satisfaction Index [39]. The survey methodology uses a psychometric “voice-of-the-customer” technique to assess consumer drivers of satisfaction (look and feel, navigation, site information, site performance, and task processes) and prioritize areas of improvement. In the CXA model, scores are based on data from randomized voluntary Web-based surveys and are reported on a scale of 0 to 100, indicating less to more customer satisfaction. Multiple item measures are combined algorithmically to compile a satisfaction index each time an adequate quantity of data has been collected through completed surveys [40]. The survey tool for the My Health*e*Vet portal includes standard questions, to allow for trend analysis of core components such as overall satisfaction, and user experience of navigation. The inclusion of custom questions on an as-needed basis further enables the collection of rich data about user demographics, needs, and preferences to address specific and time-sensitive evaluation topics and to inform ongoing design and development efforts.

The CXA survey is conducted with all veterans using My Health*e*Vet and is, therefore, a nationwide sample of veteran My Health*e*Vet users. The survey is implemented on the My Health*e*Vet portal as a Web-based pop-up browser window inviting a random sample of site visitors to participate. A persistent cookie prevents site visitors who received the survey invitation from being invited again for 90 days. When visitors accept the invitation, the survey presents when they leave the site. The loyalty factor, currently 4 pages, ensures that respondents have experienced multiple pages on the site before being prompted to participate in the survey. The sampling percentage, set at 13% in FY2008 and later changed to 4% in FY2010 due to the large amount of data being collected and increasing survey completion rates, ensures that a minimum number of site visitors are surveyed in order to reduce respondent burden while enabling the collection of adequate data.

This paper presents selected analyses of the CXA survey data collected over a course of 10 years, including a comparison of data collected early in the implementation of My Health*e*Vet (FY2008) to more recent data (FY2017), to examine the characteristics of patient portal users and their preferences.

Data analysis is primarily descriptive and based on forced-choice responses. Analysis of open-ended comments includes a combination of traditional qualitative techniques [41,42] along with keyword clustering to group related comments for further analysis. A variety of strategies are used to then translate insights into iterative improvements, including ongoing data reviews, requirement elaboration, design sessions with key stakeholders, and review of user feedback after deployment of enhancements.

## Results

### Overview

We first present a recent summary of user demographics and characteristics and patterns of portal use and relevant comparisons to previous data. Following our case study approach, we then provide selected examples from the My Health*e*Vet evaluation program to illustrate how different assessments that capture the voice of the customer have directly informed the evolution of the portal and the addition of new functionality. For FY2008 (October 1, 2007-September 30, 2008), of the surveys presented to site visitors, 17.1% (100,069/585,039) were completed. For FY2017 (October 1, 2016-September 30, 2017), of the surveys presented to site visitors, 68.9% (100,555/146,023) were completed. As completion rates increased over the last decade, the sampling rate was reduced in FY2010 from 13% to 4% in order to minimize respondent burden.

### User Demographics and Characteristics

**Figure 1:**
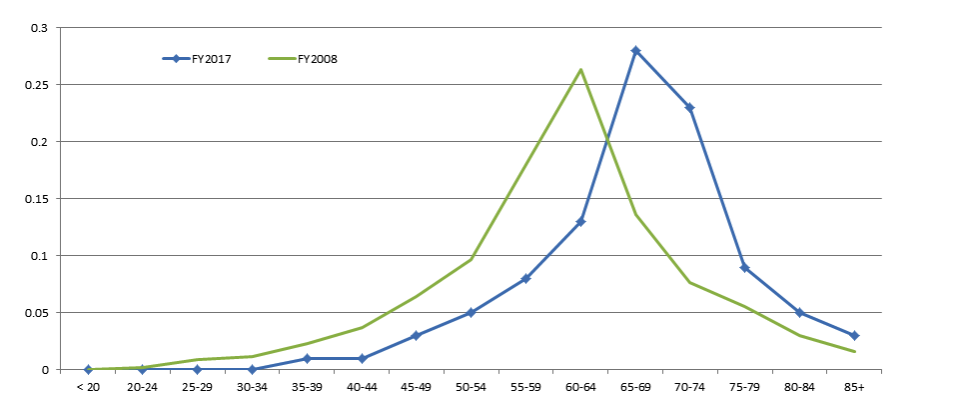
User age ranges for fiscal year (FY)2008 and FY2017.

Table 1 provides a comparison of user demographics and characteristics for all survey respondents in FY2017 and FY2008. In FY2017, 97% (97,538/100,555) of respondents were veterans compared with 93% (93,064/100,069) in FY2008. Respondents reported having completed higher levels of education, with 40% (39,990/99,974) being college graduates, completing some postgraduate school, or having a graduate or professional degree in FY2017 compared with 34% (732/2152) in FY2008. The proportion of male respondents increased slightly to 93% (90,507/97,319) in FY2017. In FY2017, respondents were generally older, with 64% (59,819/93,467) in the age range of 60-74 years compared with 47% (14,563/30,984) in FY2008; furthermore, 17% (15,889/93,467) of respondents in FY2017 were older than 75 years. This shift in age is also shown in Figure 1.

While 60% (60,042/100,069) of users in FY2008 reported their period of military service as the Vietnam War, this increased to 67% (67,372/100,555) of users in FY2017. Fewer users self-reported their internet ability as advanced in FY2017 (32,235/53,725, 60%) than in FY2008 (37,848/55,658, 68%), whereas more users reported it as intermediate in FY2017 (19,341/53,725, 36%) than in FY2008 (16,141/55,658, 29%). A greater proportion of respondents reported better health in FY2017, with 34% (33,323/98,007) reporting fair or poor health in FY2017 compared with 39% (15,723/40,315) in FY2008. Although the FY2008 survey did not ask users about health conditions, responses in FY2017 revealed a high prevalence of chronic conditions, including high blood pressure (15,045/22,795, 66%), high cholesterol (14,133/22,795, 62%), arthritis (13,677/22,795, 60%), chronic pain (10,714/22,795, 47%), diabetes (8434/22,795, 37%), stomach or gastrointestinal problems (8434/22,795, 37%), and heart problems (8434/22,795, 37%).

**Table 1:** Demographics and characteristics. FY: fiscal year. N/A: Not applicable. VA: Department of Veterans Affairs.

Type		FY2017	FY2008
**Role^a^, n (%)**		**100,555**	**100,069**
	Veteran	97	93
	Family member	3	5
	Veteran Service Organization	1	1
	National Guard or Reserve	1	N/A
	General public	1	<1
	Other role	1	1
	VA employee	1	1
	Non-VA federal employee	<1	1
	Caregiver (other than family)	<1	N/A
	State or local government	<1	N/A
	Active duty	<1	<1
	News Media	N/A	<1
**Highest level of education, n (%)**		**99,974**	**2154**
	Did not complete high school	3	2
	High school graduate	13	17
	Some college or vocational school	44	44
	College graduate	21	19
	Some postgraduate school	6	5
	Graduate or professional degree	13	10
**Health conditions^a^, n (%)**		**22,795**	**N/A**
	High blood pressure	66	N/A
	High cholesterol	62	N/A
	Arthritis of any kind	60	N/A
	Chronic pain	47	N/A
	Diabetes	37	N/A
	Stomach or gastrointestinal problems	37	N/A
	Heart problems	37	N/A
	Mental health or psychiatric condition	34	N/A
	Cancer of any kind	29	N/A
	Lung problems (including asthma)	25	N/A
	Neurological disorders	13	N/A
	Other	12	N/A
	Prefer not to answer	2	N/A
**Age, n (%)**		**93,467**	**30,984**
	Under 20	<1	<1
	20-24	<1	<1
	25-29	<1	<1
	30-34	<1	1
	35-39	1	2
	40-44	1	4
	45-49	3	6
	50-54	5	10
	55-59	8	18
	60-64	13	26
	65-69	28	14
	70-74	23	7
	75-79	9	5
	80-84	5	3
	85 or older	3	1
**Gender, n (%)**		**97,319**	**31,020**
	Male	93	91
	Female	7	9
**Self-reported health status, n (%)**		**98,007**	**40,315**
	Excellent	4	5
	Very good	21	18
	Good	41	38
	Fair	27	29
	Poor	7	10
**Self-reported internet ability, n (%)**		**53,725**	**55,658**
	Beginner	4	4
	Intermediate	36	29
	Advanced	60	68

^a^Multiple categories may be selected.

### Portal Access Patterns and Usage

Table 2 provides a summary of survey respondents’ self-reported portal access patterns and usage. While the proportion of survey respondents who use VA health care services remained the same from FY2008 to FY2017 (96%), in FY2017 50% (47,066/94,132) of respondents stated that they also use a community non-VA provider. When asked about travel time to the nearest VA facility, 32% respondents reported it to be less than 30 minutes in both FY2008 and FY2017; however, a greater proportion noted fewer minutes of travel time in FY2017 than in FY2008. For example, 37% (19,902/53,788) respondents in FY2008 reported a travel time greater than 60 minutes to the nearest VA facility compared with 24% (12,075/50,313) in FY2017. The proportion of respondents who have a Premium account, offering them access to all portal services, increased significantly from 60% (56,884/94,806) in FY2008 to 77% (73,001/94,806) in FY2017. While a greater number of respondents were first time users in FY2008 (12,074/100,617, 12%) than in FY2017 (4022/100,555, 4%), respondents reported using the portal more frequently in FY2017, with 46% (45,255/100,555) using it about once a month and 29% (29,161/100,555) using it about once a week. When asked about the length of use in FY2017, 63% (63,349/100,5455) respondents reported having used My Health*e*Vet for more than 2 years.

### User Preferences and Responsive Design

In keeping with the UCD process, VA has used direct veteran feedback about preferences obtained via the CXA survey to shape the identification and prioritization of portal improvements. In this section, we describe how different types of user feedback have directly informed enhancements to the core components of the system including available features, interface design, content, policy, and prospective functional design of a new feature.

#### Additional Services Desired

UCD principles focus on identifying what features users consider to be essential. One survey question that has been crucial in getting feedback to prioritize portal enhancements over the last decade has been “What additional services would you like to see on My Health*e*Vet?” As shown in Table 3, additional services desired by users in FY2008 included the ability to view (79,892/92,160, 87%) or schedule (68,395/92,160, 74%) VA Appointments, access information from the VA medical record (67,714/92,160, 73%), and Web-based secure communication with my doctor (58,878/92,160, 64%). Each of these features was subsequently added to the portal (Table 4).

Secure Messaging implementation began in 2008, which enabled secure Web-based communication with VA health care teams, with the full national release to all VA primary care providers in 2012. Veterans could then also use Secure Messaging to request VA Appointments. The ability to view upcoming VA Appointments was deployed in 2011, with appointment email reminders added in 2015. Building on early access to VA Medication History, VA incrementally expanded the types of information from the VA medical record available in My Health*e*Vet, for example, preventative Wellness Reminders (2009), VA Chemistry or Hematology Lab Results (2011), VA Immunizations (2012), VA Notes including mental health notes (2013), a more comprehensive Medication List that includes patient-reported non-VA medications (2016), Surgical and Clinical Procedure Notes (2017), and VA Medical Images and Reports (2017).

Additional services desired by users in FY2017 included the ability to schedule or change VA Appointments directly (52%), a list of health care providers and their contact information (44%), a tool to determine whether different medications are safe when taken together (26%), and the ability to view and pay VA bills or copayments (25%). The ability to schedule or change VA Appointments directly was piloted in FY2017 and is being rolled out to all VA facilities in FY2018. The enhanced VA Health Summary (2017) provides VA patients with a list of their primary health care providers, which will be expanded in FY2018 to include their contact information. Although VA has not yet invested in the development of tools to check medications for potential interactions; this enhancement is being given further consideration in FY2018. In addition, the ability to view a VA Patient Statement and remit payment is also being developed and scheduled for pilot testing in FY2018.

#### Patient-Identified Main Improvements

In addition to eliciting user feedback on additional services desired, the CXA survey also invites open-ended comments in response to the question: “What is the main improvement that you would suggest for the My Health*e*Vet website?” Below we offer examples of how these comments have led to user-directed improvements.

With the expansion of Lab and Test Results and the addition of VA Notes in January 2013, one theme that surfaced in the ensuing months was veterans’ desire for more timely access to this information. These comments were crucial in driving VA policy change to reduce the hold period for lab results and progress notes from 7 calendar days after verification to 3 calendar days. This policy change was implemented in June 2013.

To complement the prioritization of known desired additional services by users, open-ended main improvement comments also allow veterans to suggest needed functional enhancements in their own words. In October 2013, thematic analysis of free-text comments identified the need for multiple functional enhancements including the ability to track delivery of the filled prescriptions, the desire to be notified before automatic log out when the user session was nearing time-out, and the need for improved navigation to complete common tasks. The ability to track delivery of mailed prescriptions by opting-in to receive an email notification was deployed in 2015. Other functional enhancements (session time-out warning, improved navigation, and reduced number of steps to complete common tasks) became core requirements for a major website redesign project. The session time-out warning and ability to extend the session time was deployed as VA migrated to a content management system in October 2016. The incremental deployment of website redesign in October 2016 and September 2017 was significantly informed by veteran main improvement comments:

Publishing labs and notes within 24 hours of a lab or health visit. Waiting a week for lab results, or a week for Dr and nurse notes is absurd, given that the health problem is “right now,” not right now + seven days, especially when Dr's notes are also instructions for post visit procedures, such as when and how much meds to take, or “If it hasn't improved in three days” see me. Not everyone is “present” at the end of a visit due mostly to anxieties surrounding the visit.

**Table 2:** Access patterns. FY: fiscal year. N/A: not applicable. VA: Department of Veterans Affairs.

Respondent Characteristic		FY2017	FY2008
**Use VA health care services, n (%)**		**98,007**	**29,528**
	Yes	96	96
	No	3	4
	Not sure	1	N/A
**Use community non-VA providers, n (%)**		**94,132**	**N/A**
	Yes	50	N/A
	No	47	N/A
	Not sure	3	N/A
**Premium My HealtheVet account^a^, n (%)**		**94,806**	**100,617**
	Yes	77	60
	No	9	24
	Not sure	15	15
	Not applicable	N/A	1
**Travel time to nearest VA facility^a^, n (%)**		**50,313**	**53,788**
	Less than 30 min	32	32
	30-60 min	43	32
	61-90 min	14	20
	91 min to 2 h	6	9
	Over 2 h	4	8
	Not sure	N/A	1
**Frequency of use, n (%)**		**100,555**	**100,617**
	Daily or more than once a day	5	5
	About once a week	29	25
	About once a month	46	49
	About every 6 mo	9	5
	Less than every 6 mo	4	3
	First time	4	12
	Not sure or Do not recall	2	N/A
**Length of use, n (%)**		**100,555**	**N/A**
	Less than 6 mo	9	N/A
	6 mo-less than 1 y	6	N/A
	1-2 y	19	N/A
	More than 2 y	63	N/A
	Not sure or Do not recall	3	N/A

^a^Percentages do not add to 100 due to rounding.

**Table 3:** Additional services desired. FY: fiscal year. VA: Department of Veterans Affairs.

Service		n (%)
**FY2017**		**88,308**
	Schedule or change my VA appointments	45,695 (52)
	View a list of my VA health care providers and their contact information	38,489 (44)
	Check to determine if my different medications are safe taken together	22,710 (26)
	View or pay my VA bills or copayments	21,768 (25)
	Use a mobile app for My Health*e*Vet	13,823 (16)
	Advance check-in for my VA clinic visits	12,677 (14)
	Authorize sharing information with my Non-VA health care provider	11,467 (13)
	Authorize sharing information with my VA health care team	8851 (10)
	Authorize sharing information with other people (eg, family, caregiver)	7584 (9)
	Other	6573 (7)
	More Web-based educational programs	5396 (6)
	Join a Web-based forum to discuss health issues with other veterans	3831 (4)
**FY2008**		**92,160**
	View my upcoming appointments	79,892 (87)
	Schedule or change my appointments	68,395 (74)
	Look at information in my VA medical record	67,714 (73)
	Web-based, secure communication with my doctor	58,878 (64)
	Checking that different medications I take are safe when used together	45,986 (50)
	Reminders of preventive care I need (eg, shots, cancer screening)	34,707 (38)
	Notification of new content or features on the site	32,418 (35)
	Advance check-in for my VA clinic visits	31,863 (35)
	Monthly email newsletter	24,186 (26)
	Share information that I have stored in My Health*e*Vet with other people	23,088 (25)
	Advanced directive (eg, living will, durable power of attorney)	20,418 (22)
	Educational programs	18,800 (20)
	Information about the quality of VA health care	11,231 (12)
	Other	8791 (10)

**Table 4:** My Health*e*Vet history and feature enhancement milestones. DoD: Department of Defense. EHR: electronic health record. VA: Department of Veterans Affairs.

Year	Milestone
1999	My Health*e*Vet Pilot at 9 VA Medical Centers
2003	National My Health*e*Vet Portal deployed
2004	New user registration module deployed Expansion of self-entered data modules
2005	Prescription (Rx) Refill requests Additional self-entered modules
2006	In Person Authentication to Upgrade to Premium Account
2007	Account Activity History Forgot User ID and Password Support Upgraded Health Calendar
2008	Secure Messaging deployed for voluntary provider use Master Veteran Index synchronization
2009	VA Wellness Reminders
2010	VA Blue Button Feature (Download My Data)
2011	VA Appointments VA Allergies VA Chemistry and Hematology Lab Results DoD Military Service Information Display Rx Medication Name
2012	Secure Messaging with all VA primary care providers VA Immunizations Veterans with DoD log-on credential can use to log in to portal (single sign on) Social media content promotion
2013	Expansion of VA EHR data in VA Blue Button Report (eg, VA Notes, VA Radiology Reports, Pathology Reports, Microbiology Lab Results, etc) Basic VA Health Summary added Hold Periods reduced from 7 to 3 calendar days HealtheLiving Assessment (health risk appraisal) Veterans Health Library
2014	Ability to send Secure Messaging attachments Migration to cloud environment for system stability, scalability, and performance Log-in enhancements Display medication images in pharmacy module
2015	Secure Messaging Workload Credit Rx Refill Shipment Email Notification VA Appointment Email Reminders Save Secure Messaging Progress Notes to VA EHR Subscribe to My Health*e*Vet Newsletter
2016	Content Management System deployed Incremental Redesign: homepage dashboard navigation Session time out warning
2017	Enhanced VA Health Summary with Surgical and Clinical Procedure Notes Incremental Redesign theme deployment VA Medical Images and Reports Pilot Personalized Veteran’s Benefits Handbook Appointment scheduling

**Figure 2:**
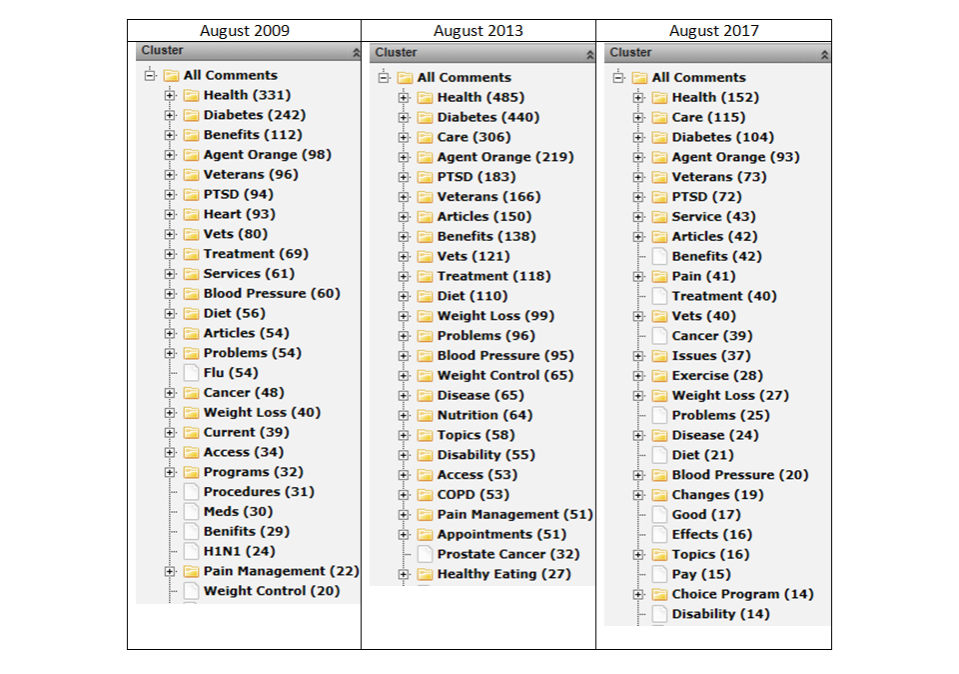
Veteran comments about hold periods.

Why does it take so long for results of lab work, radiology, notes from Drs to show up? It can take a week or more. My Dr already called with the results yesterday but I still can’t see it here. Also saw GI Dr 3 days ago and not notes here. I wish we could access our information sooner.

Eliminate wait period to view VA Notes, Results, etc. Once the provider has entered the note or viewed the results of test, they should be made available for viewing by the veteran.

#### Aligning Content With Patient-Suggested Topics of Interest

Periodically, an open-ended question is added to the survey asking users about topics of interest for portal content, such as feature articles to ensure that content is directly aligned with veterans’ needs and preferences. An editorial calendar is created to provide articles throughout the year focused on these topics. Topics are also highlighted in a subscription-based monthly electronic newsletter that was developed in 2015 as a user-desired additional service (see Table 3), with more than 500,000 subscribers in FY2017. Examples of topic clusters for August 2009 (N=1809), August 2013 (N=3300), and August 2017 (N=1189) are shown in Figure 2. In 2017, the top user-suggested topics included Health (“general health, age concerns, pre-existing medical issues”), Diabetes (“articles on diabetes and feet or hand or finger neuropathy”), Care (“information on special health care programs for specific conditions”), and Agent Orange (“need more information on Agent Orange exposure and health issues”).

#### Prospective Functional Design of a New Feature

Veteran feedback has also driven the functional design of new features. One feature that is currently being developed is the ability for the users to assign a delegate who can access their account. For example, a spouse or caregiver who may be assisting a veteran patient in managing his or her health. In October 2014, VA convened key stakeholders and subject matter experts to define the business requirements for this feature; however, there was a lack of consensus on a key functional requirement: whether “read-only” access should allow or restrict a delegate’s ability to also print and download data. Using the CXA survey, veterans were asked “If you approve read access for another person to help you manage your personal health information, what would you want that person to be able to do?” 

Of those veterans with a preference to delegate read access to another person, 75% (8194/11,006) would want such access to include print and download capability, while 14% (1541/11,006) would want a delegate to be able to read or view their information on the screen, but not print or download it. With this direct veteran input on desired functional design, requirements were prospectively aligned with user preferences. Data were also collected to assess patient preferences regarding delegating access to health information [43], use of My Health*e*Vet to transfer information [44], how veterans with non-VA providers use the Blue Button feature to share information with their non-VA providers [45], and the veteran experiences with access to their VA Notes [36].

### Website Redesign and Satisfaction Trends

Analysis of CXA data over the course of the last decade has been an integral part of the recent My Health*e*Vet website redesign initiative by enabling a deeper understanding of the end users and their tasks and goals, in keeping with UCD principles. As shown in Table 5, while 75% (75,241/100,617), 24% (23,923/100,617), and 18% (17,899/100,617) users in FY2008 accessed the portal to request a prescription refill, view their medication history, and look up information about a medication, respectively, user goals and tasks in FY2017 have shifted and expanded. Although prescription refill requests remained a predominant task (53,193/100,555, 53%), users also accessed the portal to view their VA Appointments (38,664/100,555, 38%), communicate with their health care team using Secure Messaging (28,952/100,555, 29%), track the delivery status of their medication refills (23,884/100,555, 24%), view their lab or test results (19,382/100,555, 19%), and access their VA health records (11,966/100,555, 12%). An important goal of the culminating website redesign was to improve navigation and usability for these specific core features, and the overall customer satisfaction index score was used as a performance indicator.

**Figure 3:**
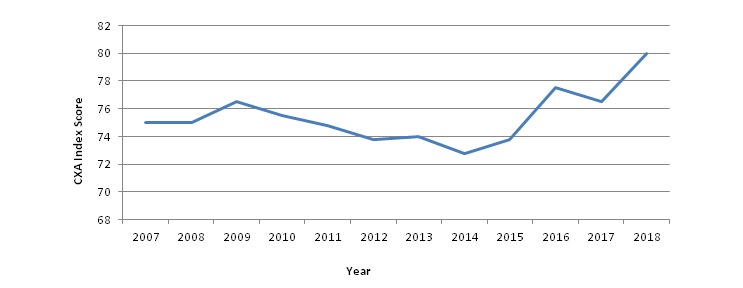
Open-ended comment clusters for topics of interest.

Historical customer satisfaction trends are shown in Figure 3. From October 2007 to October 2015, the aggregate average CXA score was 74, based on 945,480 completed surveys. The average for the 12 months that followed was stable at 76 (N=139,934). While multiple factors impacted customer satisfaction over the last decade, including a period of system performance issues in 2014 that was resolved by improving system architecture, the overall trend toward greater customer satisfaction is evident.

**Figure 4:**
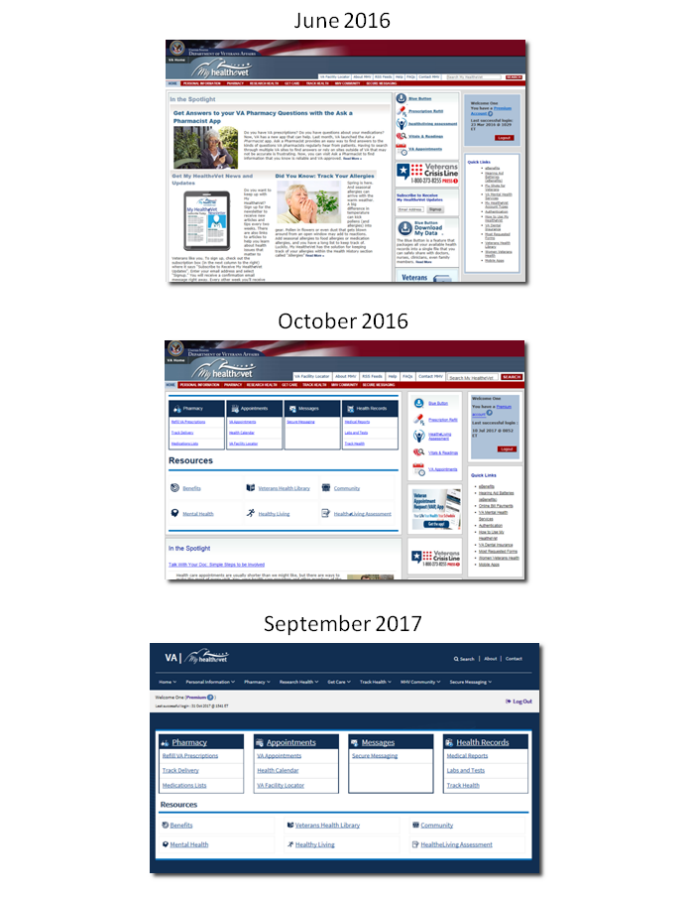
Historical customer satisfaction trends. CXA: customer experience analytics.

In October 2016, as part of an incremental website redesign, a dashboard was added to the portal home page to enhance user access to the core features (Figure 4).

**Figure 5:**
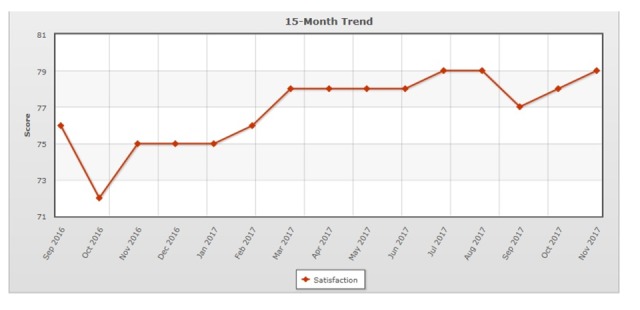
Incremental changes to My HealtheVet home page.

As anticipated, the introduction of changes to the website resulted in an initial decrease in satisfaction (72), followed by satisfaction recovery (75), and subsequent increase to a new high of 79 (Figure 5). A similar pattern was observed with the deployment of additional website redesign changes in September 2017. Satisfaction initially decreased (77), but then recovered to previous levels (79). Satisfaction continued to increase in January 2018 (80).

**Table 5:** User-specified goals and tasks. FY: fiscal year. N/A: not applicable. VA: Department of Veterans Affairs.

Reason for visit or goal trying to accomplish^a^	FY2017 (N=100,555), n (%)	FY2008 (N=100,617), n (%)
Request a prescription refill	53,193 (53)	75,241 (75)
View my VA Appointments	38,664 (38)	N/A
Use Secure Messaging to communicate with my VA health care team	28,952 (29)	N/A
Track the status of my prescription refill delivery	27,516 (27)	N/A
View my medication history	23,884 (24)	23,923 (24)
View my lab or other test results	19,382 (19)	N/A
Access my VA health records or Blue Button or VA Health Summary	11,966 (12)	N/A
View my VA Notes (written by my health care team)	11,058 (11)	N/A
Look up information about a health condition or medication	9393 (9)	N/A
Learn more about features that are available	9149 (9)	N/A
Look up information about a medication	N/A	17,899 (18)
Find information about VA benefits	9111 (9)	6246 (6)
Enter or keep track of personal information	5695 (6)	14,507 (14)
Other	5101 (5)	9198 (9)
Enter or keep track of personal health care information (eg, blood pressure)	3202 (3)	13,125 (13)
Use the Veterans Health Library (Research a health condition)	2648 (3)	6367 (6)
Enter information about my non-VA medications or supplements	2288 (2)	N/A
Find a VA facility	1646 (2)	2206 (2)
Complete a HealtheLiving Assessment	1533 (2)	N/A

^a^Multiple categories may be selected.

**Figure 6:**
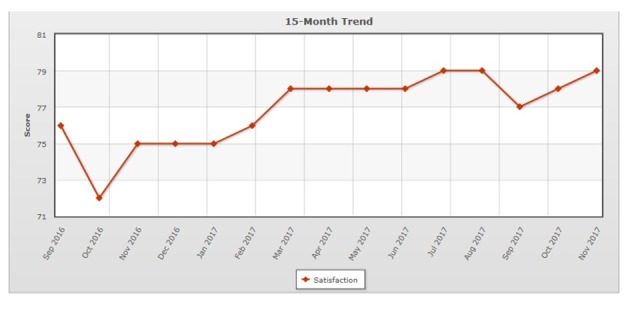
Customer experience analytics customer satisfaction index 15-month trend.

## Discussion

### Principal Findings

The literature on adoption and use of patient portals highlights the need for health care organizations to employ UCD approaches to ensure that portals align with end users’ characteristics, needs, preferences, and goals, and, ultimately, help advance portal implementation. In its commitment to UCD, one method that VA has used to accomplish the above is a continuous survey to elicit a direct feedback from a random sample of veterans who use VA’s patient portal, My Health*e*Vet. In combination with other methods, such as targeted research studies, the CXA survey has enabled a deeper understanding of portal users and directly informed changes in portal features, functions, policies, and processes. By incorporating the results of this systematic evaluation of the user experience into the portal redesign, VA aims to continue to enhance the ability of My Health*e*Vet to engage and activate veterans in managing their health.

### Patient Portal Users

This study compared the characteristics and behaviors of users during the early period of patient portal implementation, 2008, with that of later adopters. This provided a trajectory of how portal use has evolved over a decade. Many aspects remained stable, while others showed clear trends toward portal adoption by populations believed less likely to use patient-facing health technologies. While only 13.4% (89,780/670,000) portal users in FY2008 were VA patients with a Premium account, by FY2017, this increased to 62.5% (2.5 million/4 million) users. Despite early assumptions about older users not adopting and using patient portals [46,47], leading to a gray digital divide [48], the VA experience reveals an increasingly elderly population of users. Within the veteran population, research has shown that VA patients tend to be older and more socioeconomically disadvantaged than veterans who do not rely on VA for care [49]. Although the survey indicates that the majority of users have one or more chronic health conditions and access the portal with increasing frequency, the survey results also suggest a trend toward those with less internet ability and better health also accessing the patient portal. This trend may be a result of the portal expanding the types of transactional services that users find convenient, based on direct veteran input. It also suggests that the portal is engaging a broader segment of the veteran population. Although the proportion of female veterans responding to the survey decreased slightly in FY2017 (from 9% to 7%), the overall population of female veterans was estimated to be 9.4% in 2015. However, only 22.4% used VA health care services [50], which is a key driver for accessing the patient portal. Portal users in FY2017 also tended to have completed higher levels of education than those in FY2008. This may be reflective of changes in the veteran population overall, with the enhanced provision of educational support programs for separating service members. Given that half of the survey respondents in FY2017 reported that they also use community non-VA care providers, VA will need to continue to develop tools that enable effective information sharing across settings of care. Portal functions that support consumer-mediated health information exchange are currently in early field testing [51]. These patient portal user trends align with similar trends for the VA patient population overall in terms of gender (91% male), age (median age of male VA patients, 64 years), and increasing use of VA education benefits [52].

### Incremental Portal Redesign

Based on user self-report about goals and tasks, a significant redesign of the website was undertaken to enhance navigation to the features aligned with the most common user tasks and to decrease the number of steps to accomplish these. After an initial period of satisfaction decline, anticipated due to the phenomenon of change aversion [53], the satisfaction index recovered and increased. Once users adjusted to the change, they were more satisfied with the new design as measured using the CXA satisfaction index. Looking ahead, there are additional improvements and enhancements that will be important to address.

### Limitations

It is important to note that the results of the CXA survey reflect the characteristics and perspectives of a random sample of portal users who are invited and opt to participate in the survey and may not be fully generalizable to the larger population. More broadly, the respondent sample represents patient portal users; therefore, other methods are also needed to elicit input from veterans who are not portal users to understand their characteristics and preferences and identify barriers that may exist to system access and use. VA is in the process of adding questions to its patient experience survey, administered to veterans who had a recent medical encounter, to help fill this gap, and ongoing research about veteran preferences for digital tools and services provides complementary insights [32,33]. There may also be data that were not collected in the survey that could be important. Since the survey is anonymous, there is no opportunity to follow up with respondents for more information or clarification. Despite limitations inherent to an anonymous survey, it has the benefit of enabling a continuous flow of direct feedback. While the findings from our case study may not be fully generalizable to other patient populations, the principle of using agile approaches to employ UCD has potential to be a promising implementation strategy for other health care organizations.

### Conclusions

By leveraging UCD principles, VA has continued to enhance its patient portal and supported its continued implementation, achieving significant growth in adoption and use over the last decade. While quantitative and qualitative research studies are an important component of patient portal evaluation, more agile methods are also needed to complement formal research efforts. As illustrated through this case study, we have found the ongoing administration of a continuous voluntary Web-based survey as an efficient and effective way to capture veteran’s voices about who they are, how they use the patient portal, what improvements are needed, and what additional services are desired. This approach, together with others intended to explore the perspectives of veterans who are not portal users, will help ensure that VA’s health information technology services are developed and enhanced to optimize the benefits to all VA patients. With impending changes to VA’s EHR platform, capturing veteran’s voices is more crucial than ever. More broadly, developing patient portals as an effective patient engagement strategy will require that UCD principles are employed to foster adoption and sustained use. In an era of finite resources, leveraging the “voice-of-the-customer” techniques helps ensure that the portal continues to meet patients’ needs in ways that enhance full participation in their own health care.

## Abbreviations

CXA: customer experience analytics
EHR: electronic health record
KP: Kaiser Permanente
UCD: user-centered design
VA: Department of Veterans Affairs
